# Discovering reference-missing cell types in bulk transcriptomics

**DOI:** 10.1093/bioinformatics/btag412

**Published:** 2026-08-21

**Authors:** Yimin Fan, Yixuan Liu, Yunhua Zhong, Yue Wang, Kin Hei Lee, Yixuan Wang, Xinyuan Liu, Jiayi Li, Xuesong Wang, Ziqian Lin, Lei Li, Yu Li

**Affiliations:** Department of Computer Science and Engineering, The Chinese University of Hong Kong, HKSAR, Hong Kong, China; Department of Computer Science and Engineering, The Chinese University of Hong Kong, HKSAR, Hong Kong, China; Department of Computer Science and Engineering, The Chinese University of Hong Kong, HKSAR, Hong Kong, China; Department of Computer Science and Engineering, The Chinese University of Hong Kong, HKSAR, Hong Kong, China; Department of Computer Science and Engineering, The Chinese University of Hong Kong, HKSAR, Hong Kong, China; Department of Computer Science and Engineering, The Chinese University of Hong Kong, HKSAR, Hong Kong, China; Department of Computer Science and Engineering, The Chinese University of Hong Kong, HKSAR, Hong Kong, China; Department of Computer Science and Engineering, The Chinese University of Hong Kong, HKSAR, Hong Kong, China; Department of Computer Science and Engineering, The Chinese University of Hong Kong, HKSAR, Hong Kong, China; Department of Computer Science and Engineering, The Chinese University of Hong Kong, HKSAR, Hong Kong, China; Canchen Technology, Shenzhen, China; Department of Computer Science and Engineering, The Chinese University of Hong Kong, HKSAR, Hong Kong, China

## Abstract

Bulk RNA-seq deconvolution methods rely on single-cell reference data to estimate cell-type proportions in heterogeneous tissues, but certain cell types may be systematically absent from single-cell references due to technical limitations such as poor dissociation efficiency, low capture rates, or cell fragility. While recent studies have shown that signatures of missing cell types persist in deconvolution residuals, existing approaches cannot automatically determine how many cell types are missing, estimate their specific proportions, or reconstruct their expression signatures. Here, we present DeconX, a computational framework that addresses these limitations by generating “pseudo-cells” from deconvolution residuals, enabling estimation of the number, proportions, and expression signatures of missing cell types. Through comprehensive evaluation on simulated datasets, we demonstrate that DeconX accurately recovers missing cell-type proportions and expression profiles across varying conditions, and systematically identifies key factors affecting performance, including expression similarity between missing and reference cell types and missing cell-type proportions. Application to high-grade serous ovarian cancer (HGSOC) samples reasonably identifies and quantifies adipocyte populations that are absent from matched single-cell references, recovering biologically interpretable expression signatures consistent with known adipocyte markers. We further demonstrate that DeconX can reasonably determine the number of missing cell types, supporting automated analysis of unknown tissue compositions. DeconX transforms residual-based missing cell-type inference from exploratory analysis into a complete computational framework, enabling accurate characterization of tissue heterogeneity from archival bulk RNA-seq data even when single-cell references are incomplete. The source code is available at https://github.com/Seniorious123/DeconX/, and the documentation and tutorials are available at https://deconx.readthedocs.io.

## 1 Introduction

Bulk RNA sequencing (RNA-seq) remains the primary platform for large-scale cohort studies due to its cost-effectiveness and the availability of extensive public repositories such as TCGA (Weinstein *et al*. 2013). These datasets enable population-level analyses across thousands of samples to identify disease biomarkers and predict treatment responses. However, bulk RNA-seq measures average gene expression across all cells in a tissue sample, obscuring cellular heterogeneity. Understanding the cellular composition of bulk samples is therefore critical for interpreting transcriptomic data and linking molecular signatures to specific cell types.

Cell type deconvolution aims to computationally infer the proportion and expression profile of cell types from bulk RNA-seq data by leveraging single-cell RNA sequencing (scRNA-seq) references as prior knowledge. Current methods (Chen *et al*. 2022, Wang *et al*. 2024) assume that the reference atlas contains all cell types present in the bulk samples. However, this assumption does not always hold in practice, as certain cell types may be absent from single-cell references due to technical limitations such as poor dissociation efficiency, low capture rates, or cell fragility (Ivich *et al*. 2025), as well as biological factors including rare cell populations or disease-associated cellular states that are not represented in available atlases (Joanito *et al*. 2022). When bulk samples contain cell types absent from the reference, existing methods produce biased proportion estimates or incorrectly attribute signals from missing cell types to known populations.

A recent study demonstrated that signals of missing cell types persist in deconvolution residuals and can be detected using non-negative matrix factorization (NMF) (Ivich *et al*. 2025). However, this approach requires prior knowledge of how many cell types are missing, relies on manual interpretation of NMF factors, cannot reconstruct expression profiles of missing cell types, and cannot estimate their absolute proportions in individual bulk samples. These limitations restrict the approach to exploratory analysis rather than an effective tool for systematic missing cell-type inference from bulk transcriptomics samples.

Here, we present DeconX, a deep learning framework that simultaneously performs cell type deconvolution and discovers missing cell types through iterative residual analysis. DeconX employs a deep autoencoder architecture to learn compressed representations of cellular signatures and decode them into cell type proportions. In each iteration, the framework analyzes reconstruction residuals—the portions of bulk signals unexplained by known cell types—to identify samples enriched for missing populations, extracts their gene expression signatures, and integrates them as pseudo-cells into the reference for model retraining. This iterative cycle continues until a multi-step stopping criterion determines that no further missing cell types remain. This enables fully unsupervised inference of the number, proportions, and expression profiles of missing cell types. We demonstrate DeconX’s performance through comprehensive evaluation on simulated datasets and application to real-world HGSOC tumor samples.

## 2 Methods

### 2.1 Problem definition

Let $b_{j}\in R^{G}$ denote the expression profile of bulk sample *j* (j=1,…,N) across *G* genes. The single-cell reference contains *K* observed cell types with signature matrix $S\in R^{K\times G}$, where row $s_{i}$ represents the expression profile of cell type *i*.

Standard deconvolution assumes each bulk sample is a linear combination of reference cell types:


(1) $$b_{j}=\sum_{i=1}^{K}p_{ij}s_{i}+\epsilon_{j}$$

Where $p_{ij}\geq 0$ represents the proportion of cell type *i* in sample *j*, with $\sum_{i=1}^{K}p_{ij}=1$, and $\epsilon_{j}$ is technical noise.

When *M* cell types are missing from the reference, the true model becomes:


(2) $$b_{j}=\sum_{i=1}^{K}p_{ij}s_{i}+\sum_{m=1}^{M}p_{m,j}s_{m}+\epsilon_{j}$$

Where $s_{m}\in R^{G}$ and $p_{m,j}$ denote the expression profile and proportion of missing cell type *m*, respectively. Standard deconvolution estimates ${\hat{p}}_{ij}$ using only observed cell types, yielding residuals that represent the component of the bulk signal not explained by the observed reference and may retain missing-cell-type-specific signals together with technical noise (Supplementary Notes, available as supplementary data at *Bioinformatics* online):


(3) $$\mathrm{re}s_{j}=b_{j}-\sum_{i=1}^{K}{\hat{p}}_{ij}s_{i}\approx \sum_{m=1}^{M}p_{m,j}s_{m}+\epsilon_{j}$$

*Problem statement*. Given bulk samples ${\left\{b_{j}\right\}}_{j=1}^{N}$, reference signature matrix S, and residuals ${\left\{\mathrm{re}s_{j}\right\}}_{j=1}^{N}$, we aim to: (1) infer the number of missing cell types *M*, (2) estimate their proportions $\left\{p_{m,j}\right\}$, and (3) reconstruct their signature matrix $S_{m}\in R^{M\times G}$.

*Evaluation Metrics*. To evaluate DeconX performance on simulated datasets with known ground truth, we employ two complementary metrics. For proportion estimation, we use the concordance correlation coefficient (CCC):


(4) $$\text{CCC}=\frac{2\rho \sigma_{\text{true}}\sigma_{\text{pred}}}{\sigma_{\;\text{true}}^{2}+\sigma_{\text{pred}}^{2}+{\left(\mu_{\text{true}}-\mu_{\text{pred}}\right)}^{2}}$$

Where ρ is the Pearson correlation between predicted (${\hat{p}}_{m,j}$) and true ($p_{m,j}$) proportions, and μ and σ denote mean and standard deviation, respectively. CCC accounts for both correlation and agreement, penalizing systematic bias and scale differences. For signature reconstruction, we use cosine similarity:


(5) $$\text{Sim}=\frac{{\hat{s}}_{m}\cdot s_{m}}{∥{\hat{s}}_{m}∥∥s_{m}∥}$$

Which measures directional agreement between expression profiles, ranging from 0 to 1 (the expression profile is non-negative), and is robust to scaling differences introduced by normalization.

### 2.2 Datasets and data processing

#### 2.2.1 Simulated datasets

We evaluated DeconX using simulated pseudo-bulk data generated from a large-scale COVID-19 single-cell RNA-seq dataset (Stephenson *et al*. 2021). This dataset contains over 7 80 000 peripheral blood mononuclear cells (PBMCs) from patients with varying disease severities and healthy controls. We used this well-characterized dataset to generate pseudo-bulk samples with known cell-type compositions, enabling a systematic evaluation of DeconX under controlled conditions.

#### 2.2.2 Real datasets

We further applied DeconX to bulk RNA-seq data from HGSOC samples (Hippen *et al*. 2023). This dataset contains matched tumor samples with corresponding scRNA-seq and snRNA-seq profiles. Due to technical limitations in capturing large, lipid-rich cells, the scRNA-seq reference lacks adipocytes, while the snRNA-seq reference successfully captures them. We hypothesized that adipocytes would be present in the bulk samples but absent from the scRNA-seq reference. By comparing DeconX performance when using the incomplete scRNA-seq reference versus the complete snRNA-seq reference, we could test whether DeconX detects missing adipocyte signals in deconvolution residuals. The snRNA-seq reference serves as a control condition with no missing cell types.

#### 2.2.3 Pseudo-bulk data generation

**Figure 1:**
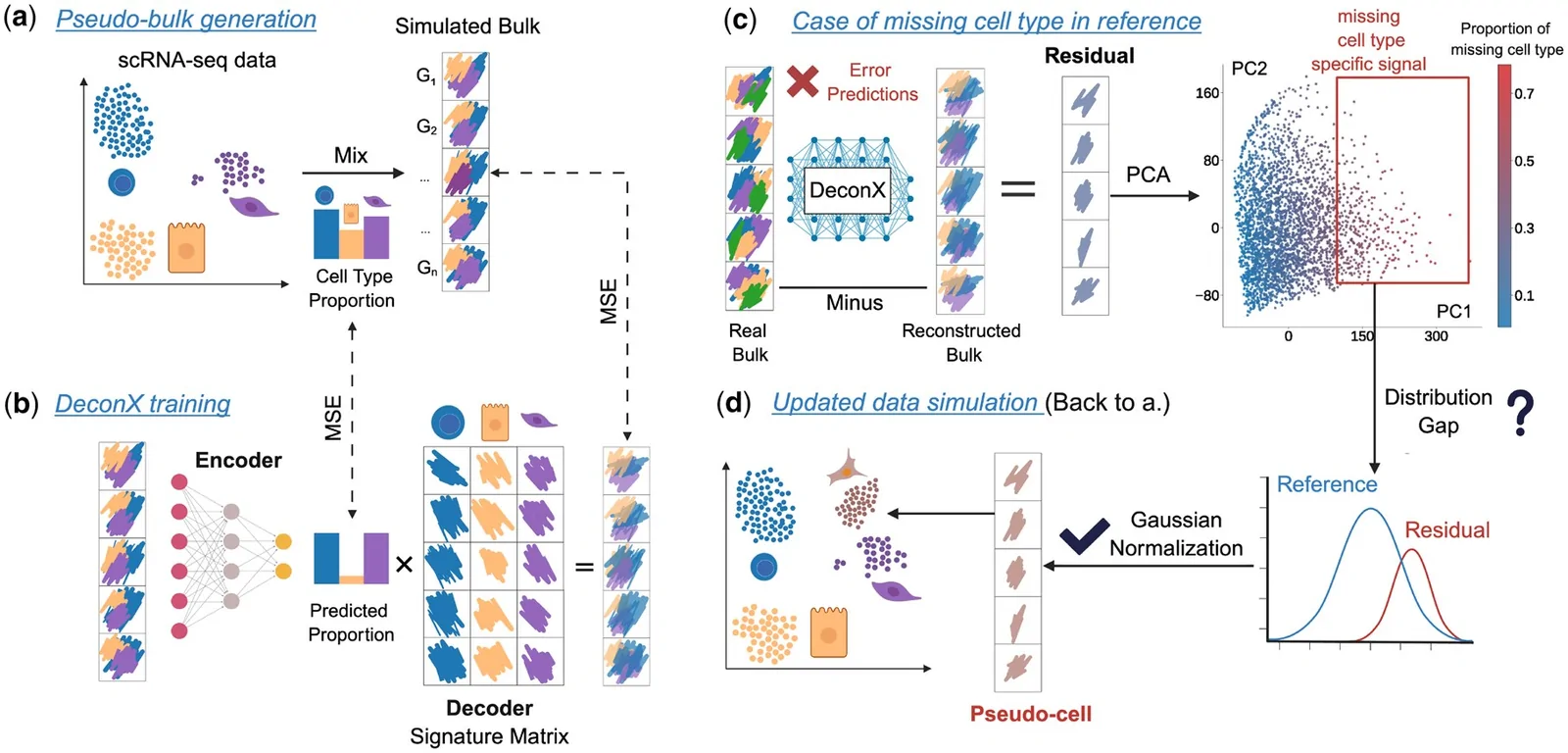
Overview of the DeconX framework, which iteratively detects and characterizes novel cell types in bulk RNA-seq deconvolution. (a) Pseudo-bulk samples are generated from scRNA-seq reference data with simulated cell type proportions. (b) An encoder-decoder architecture learns to predict proportions and reconstruct expression signatures. (c) Residuals between observed and reconstructed bulk profiles reveal missing cell type-specific signals, identified via PCA. (d) Residuals are normalized via Gaussian normalization to generate pseudo-cells, and novel cell type signatures are extracted using NNLS. These pseudo-cells are incorporated back into the reference, repeating from step a to enable iterative model refinement . The Figure is created with Biorender.com.

We generated pseudo-bulk samples by sampling cell type proportions from a Dirichlet distribution, where $\alpha_{i}$ represents the prior parameter for cell type *i* (Fig. 1a). For each sample, we randomly selected 3000 single cells according to these proportions and computed the bulk expression profile as the sum of selected single-cell profiles. In each experimental setting, we generated 600 pseudo-bulk samples for training.

### 2.3 Model architecture

DeconX employs a deep autoencoder architecture to simultaneously detect missing cell types and estimate their proportions from bulk RNA-seq residuals (Fig. 1b). The encoder-decoder structure enables an interpretable decoder that naturally represents cell-type-specific signature matrices (Chen *et al*. 2022).

The encoder maps bulk expression profiles $b_{j}\in R^{G}$ to cell-type proportion vectors $p_{j}\in R^{K}$, where *K* is the number of cell types and $p_{i,j}$ represents the proportion of cell type *i* in sample *j*. It consists of five fully connected layers (G→512→256→128→64→K) with CELU activation and dropout, followed by normalization to ensure $p_{i,j}\geq 0$ and $\sum_{i=1}^{K}p_{i,j}=1$. The decoder reconstructs bulk expression profiles using five layers (K→64→128→256→512→G) without biases, and its weights form a signature matrix $S\in R^{K\times G}$, computed as $S=\text{ReLU}(W_{1}W_{2}W_{3}W_{4}W_{5})$, where $W_{i}$ is the transpose of the *i*-th layer’s weight matrix. The reconstructed bulk expression is given by ${\hat{b}}_{j}=p_{j}^{T}S$.

Unlike black-box neural networks, DeconX’s decoder provides biologically interpretable outputs: each dimension in the latent space corresponds to one cell type, and the decoder weights directly yield gene expression profiles for these cell types. This design enables downstream analysis of cell type expression profiles using their reconstructed signature matrix $S_{m}$.

### 2.4 Optimization algorithms

DeconX employs a multi-stage training strategy to progressively discover and integrate missing cell types (Fig. 1). The core motivation is to convert bulk-level residual signals into single-cell-level representations (pseudo-cells), enabling the model to treat discovered cell types equivalently to reference cell types during retraining. This approach allows seamless expansion of the deconvolution framework without architectural redesign.

*Stage 1: Initial Training*. The model is first trained on simulated pseudo-bulk data generated from known cell types in the reference (Fig. 1a–b). The initial model $M_{0}$ is trained to minimize:


(6) $$L_{\text{init}}=\frac{1}{n}\sum_{k=1}^{n}\left[\text{MSE}({\hat{b}}_{k},b_{k})+\text{MSE}({\hat{p}}_{k},p_{k})\right]$$

Where ${\left\{b_{k},p_{k}\right\}}_{k=1}^{n}$ are the simulated pseudo-bulk samples with ground-truth proportions, and ${\hat{b}}_{k}$, ${\hat{p}}_{k}$ are the model’s reconstructed expressions and predicted proportions, respectively. This establishes a baseline deconvolution model for known cell types.

**Figure 2:**
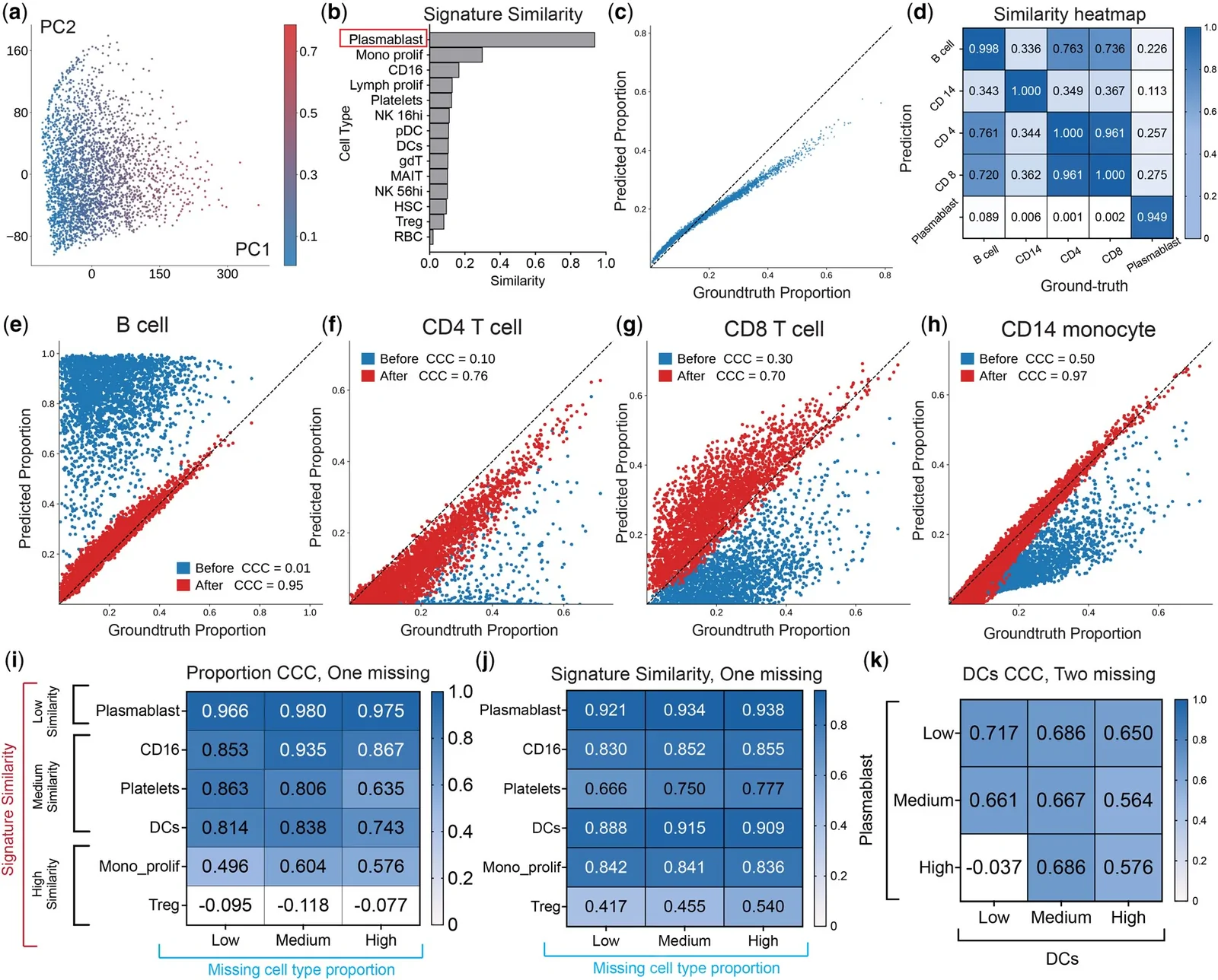
DeconX performance evaluation on simulated datasets. (a) PCA visualization of residuals $\mathrm{re}s_{j}$ colored by missing cell type proportion, showing gradient pattern capturing systematic signals. (b) Cosine similarity between inferred signature and all cell types. (c) Predicted versus ground-truth proportions of missing cell type. (d) Signature similarity heatmap between inferred and ground-truth cell types. (e–h) Proportion predictions for reference cell types before (blue) and after (red) applying DeconX to identify missing cell types: (e) B cells, (f) CD4 T cells, (g) CD8 T cells, (h) CD14 monocytes. CCC values improved substantially after incorporating the discovered missing cell type. (i-j) Impact of missing cell type proportion (x-axis) and signature similarity (rows) on: (i) proportion CCC and (j) signature similarity. (k) Proportion CCC performance for DC cell with two missing cell types at different proportion levels.

*Stage 2: Iterative Discovery and Retraining*. In each discovery round *r*, we: (1) compute residuals $\mathrm{re}s_{j}=b_{j}-M_{r-1}(b_{j})$ for all real bulk samples j=1,…,N (Fig. 1c); (2) perform differential expression analysis between residuals and reference-only pseudo-bulk data to identify discriminative genes; (3) apply PCA on the selected genes to identify samples with strong residual signals, selecting the top 10% based on PC1 scores (Fig. 1c); (4) generate pseudo-cells by *z*-score normalizing the purified residuals from selected samples to match reference data statistics, and add them to the reference as a new cell type (Fig. 1d); (5) initialize the new cell-type signature by using non-negative least squares (NNLS) to explain the mean residual of the selected samples, denoted by ${\bar{\mathrm{res}}}^{\left(r\right)}$, with the currently known signatures and taking the remaining nonnegative component as the initial signature of the newly discovered cell type (Fig. 1d). This removes residual contributions from known cell types and provides a biologically plausible initialization for the newly discovered signature.

The expanded model $M_{r}$ is retrained on newly generated pseudo-bulk samples containing both known and discovered cell types (Fig. 1a), using a two-phase schedule: first freezing parameters of existing cell types for E/3 epochs, then fine-tuning all parameters for 2E/3 epochs (E=150). The training loss incorporates additional regularization terms:


(7) $$\begin{array}{cc} L_{r}=\frac{1}{n}\sum_{k=1}^{n}\left[\text{MSE}({\hat{b}}_{k},b_{k})+\text{MSE}({\hat{p}}_{k},p_{k})\right] & \\ +\lambda_{1}∥S_{\text{known}}-S_{0}{∥}^{2}+\lambda_{2}∥W_{\text{old}}{∥}^{2} & \end{array}$$

Where $S_{0}$ denotes the initial signature matrix learned from $M_{0}$, $W_{\text{old}}$ denotes the existing decoder weights, and $\lambda_{1}=5$ and $\lambda_{2}=0.1$. Specifically, $\lambda_{1}$ constrains known cell-type signatures to remain close to their initial values, thereby mitigating drift of previously learned signatures, whereas $\lambda_{2}$ is a standard L2 regularization term on the existing decoder weights that penalizes large parameter values and helps reduce overfitting during retraining. Stage 2 is repeated for multiple rounds until the stopping criteria are met.

## 3 Results

### 3.1 Comprehensive evaluation of DeconX on simulated datasets

We first evaluated DeconX performance on simulated datasets using a case study with four cell types in the scRNA-seq reference (B cells, CD4 T cells, CD8 T cells, and CD14 monocytes) and one missing cell type (plasmablasts). We simulated pseudo-bulk samples using all five cell types but performed deconvolution using only the four reference cell types.

Visualization of residuals $\mathrm{re}s_{j}$ from simulated bulk RNA-seq data revealed a clear gradient pattern corresponding to plasmablast proportions (Fig. 2a), indicating that residuals contain distinct signals from the missing cell type and supporting the feasibility of residual-based detection. After applying DeconX, we calculated cosine similarity between the inferred cell type signature and mean expression profiles of all cell types in the dataset; plasmablasts ranked highest (Fig. 2b), confirming accurate identification. The predicted plasmablast proportions showed strong correlation with ground-truth values (Fig. 2c), and comparison between predicted and true signatures revealed high expression reconstruction accuracy with cosine similarity exceeding 0.9 (Fig. 2d).

Moreover, standard deconvolution yielded low CCC scores for reference cell types due to the presence of the unaccounted missing cell type. After DeconX identified and incorporated plasmablasts into the reference, CCC scores improved substantially for all cell types (Fig. 2e–h), demonstrating that accounting for missing cell types enhances overall deconvolution accuracy. We observed similar iterative improvements in the more challenging scenario with two missing cell types, where CCC values for reference cell types increased progressively as each missing cell type was sequentially detected and incorporated (Fig. S1, available as supplementary data at *Bioinformatics* online).

To systematically evaluate factors affecting DeconX performance, we examined two key parameters: the proportion of missing cell types and their transcriptional similarity to existing reference cell types. We tested six cell types, treating each in turn as the missing cell type, with varying signature similarities to the reference. During pseudo-bulk generation, we controlled proportions using Dirichlet parameters: α=2 for reference cell types and α∈{0.5,1,2} for the missing cell type, corresponding to low, medium, and high expected proportions (Fig. 2i–j). Results showed that DeconX achieved high accuracy (proportion CCC > 0.8, signature similarity > 0.8) across most scenarios. However, performance degraded when missing cell types were either rare (low proportion) or highly similar to reference cell types, with transcriptional similarity to the reference having a substantially greater impact on detection accuracy than cell type proportion. Regulatory T cells (Tregs), which share high transcriptional similarity with CD4 T cells, were particularly challenging to detect, yielding negative CCC values and low signature similarities. Aggregating across additional simulation scenarios revealed a strong negative correlation between confounding similarity (maximum similarity to reference cell types) and detection accuracy (Spearman r=−0.82, p<0.05; Fig. S2, available as supplementary data at *Bioinformatics* online), confirming that transcriptional distinctiveness is critical for successful detection.

We further evaluated DeconX performance in a more challenging scenario with two simultaneous missing cell types (plasmablasts and dendritic cells) across different proportion combinations (Fig. 2k). When both missing cell types were present at comparable levels, DeconX maintained robust performance with CCC values exceeding 0.8. However, when one missing cell type dominated in proportion, accuracy for the rarer cell type decreased, suggesting that highly imbalanced missing cell types pose challenges for simultaneous detection. Detailed examination revealed asymmetric effects (Fig. S3, available as supplementary data at *Bioinformatics* online): when plasmablasts dominated and DCs were rare, DC detection failed (CCC < 0.1) as their signals were masked by the abundant plasmablast signature, while plasmablasts maintained reasonable performance (CCC > 0.6) when rare due to their more distinct expression profile. These results highlight that multiple missing cell types introduce compounded challenges depending on the interplay between relative proportions, transcriptional distinctiveness, and mutual signal interference.

### 3.2 DeconX identifies adipocytes missing from the reference in real datasets

**Figure 3:**
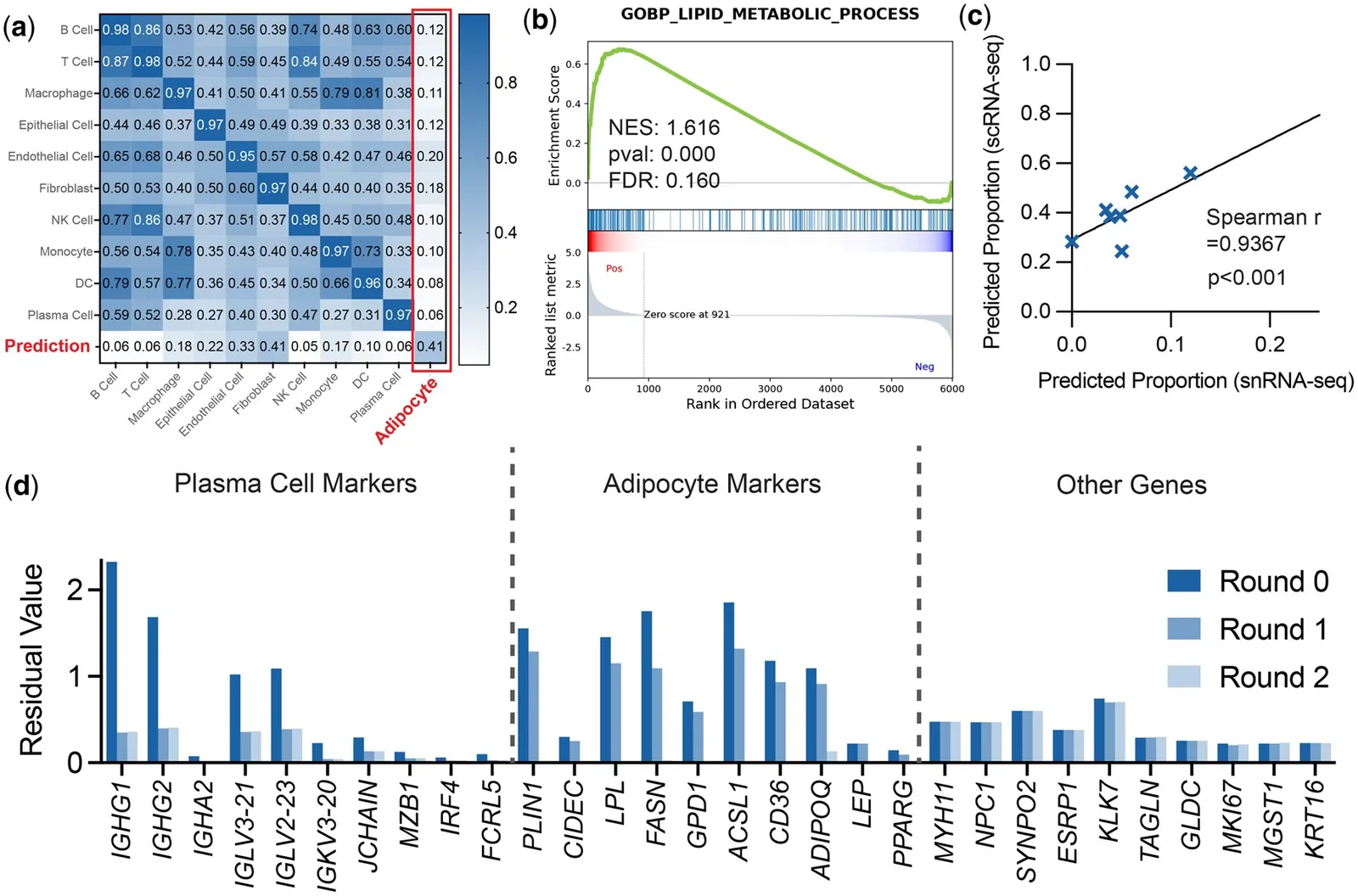
DeconX performance on real datasets. (a) Cosine similarity heatmap between the predicted signature and expression profiles of known cell types from the snRNA-seq reference. (b) Pathway enrichment analysis for genes ranked by residual expression, showing enrichment of lipid metabolic process. (c) Comparison of adipocyte proportions estimated using incomplete scRNA-seq reference versus complete snRNA-seq reference. (d) Residual expression of marker genes across DeconX iterations when both plasma cells and adipocytes are missing from the reference. Plasma cell markers, adipocyte markers, and other genes are shown separately.

To validate DeconX on real-world data, we applied it to the HGSOC dataset where adipocytes represent a technically reference-missing cell type: they were absent from the scRNA-seq reference but present in both bulk RNA-seq samples and the snRNA-seq reference. The predicted signature showed substantial similarity (cosine similarity = 0.41) to the adipocyte expression profile derived from matched snRNA-seq data (Fig. 3a), demonstrating reasonable signature recovery despite reference incompleteness. The cosine similarity was lower than that observed in simulated datasets, likely due to batch effects between bulk sequencing and single-cell sequencing platforms. We further confirmed that the predicted signature accurately recovered key adipocyte-associated biological pathways. Using gene rankings derived from the residuals, we systematically tested 1883 gene sets and identified six significant positively enriched terms at FDR <0.25 (Fig. 3b and Fig. S5, available as supplementary data at *Bioinformatics* online), such as lipid metabolic process, triglyceride metabolic process, monocarboxylic acid metabolic process, and organic acid metabolic process, which are key characteristics of adipocyte function.

We next evaluated whether the proportions of missing cell types predicted by DeconX using the incomplete scRNA-seq reference correlated with those estimated using the complete snRNA-seq reference. Adipocyte proportions estimated by DeconX with the scRNA-seq reference showed strong correlation (Spearman correlation = 0.9367) with those obtained using the complete snRNA-seq reference (Fig. 3c). However, we observed that DeconX consistently predicted higher adipocyte proportions when using the incomplete scRNA-seq reference compared to the snRNA-seq reference. We hypothesize that this overestimation occurs because the residuals contain not only adipocyte signals but also other sources of variation, such as batch effects and technical noise. These confounding signals may dilute the adipocyte-specific signal in the residuals, requiring higher estimated proportions to compensate for the reduced signal intensity during deconvolution. We further confirmed DeconX remained robust to HGSOC epithelial subtype heterogeneity, consistently recovering adipocytes without falsely identifying epithelial subtypes as missing cell types (Supplementary Notes and Fig. S8, available as supplementary data at *Bioinformatics* online).

We further tested a more complex scenario with two simultaneously missing cell types by manually removing plasma cells from the scRNA-seq reference, in addition to adipocytes. Tracking marker gene expression in residuals across DeconX iterations revealed progressive and specific depletion of missing cell type signatures. After the first iteration identified plasma cells, plasma cell marker genes (*IGHG1*, *IGHG2*, *IGHA2*) showed significant reduction in residual expression. Following the second iteration, which identified adipocytes, adipocyte marker genes (*CIDEC*, *LPL*, *FASN*, *GPD1*) were similarly depleted from the residuals (Fig. 3d). Importantly, marker genes for cell types already present in the reference remained stable across iterations. This sequential reduction demonstrates DeconX’s capability to iteratively detect and extract multiple missing cell type signatures from real tissue data. Cosine similarities between all predicted signatures and their corresponding true expression profiles from the snRNA-seq reference are shown in Fig. S4, available as supplementary data at *Bioinformatics* online. In addition, we benchmarked DeconX against a residual NMF baseline (Ivich *et al*. 2025) on both simulated and real datasets, where DeconX showed stronger recovery of missing-cell-type signatures (Fig. S6, available as supplementary data at *Bioinformatics* online). We further evaluated the sensitivity of DeconX to the PCA top-% threshold in both real and simulated datasets, confirming its robustness (Fig. S7, available as supplementary data at *Bioinformatics* online).

### 3.3 Determination of DeconX stopping criteria

**Figure 4:**
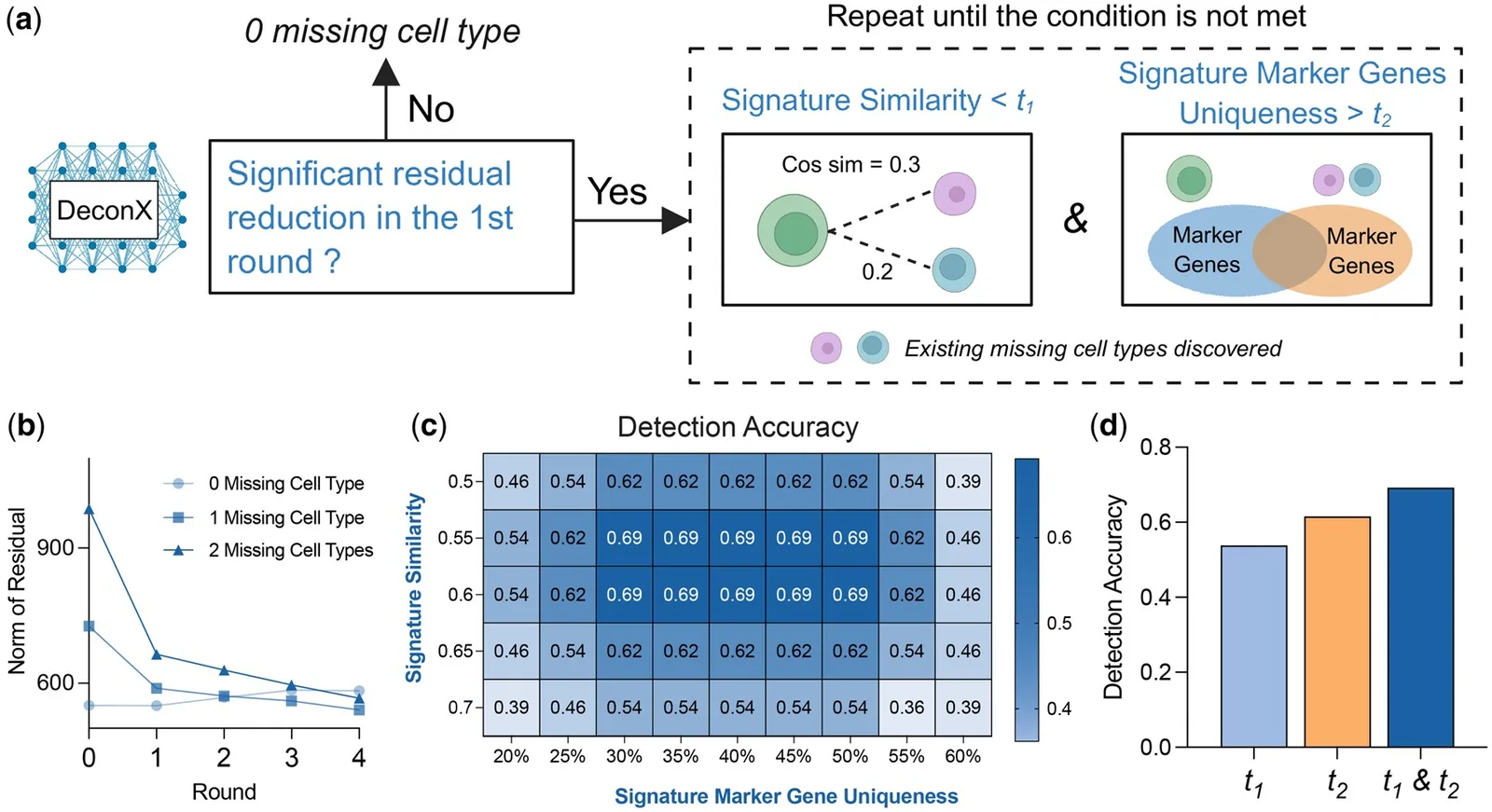
Evaluating the stop criteria for the missing cell type discovery process. (a) Schematic illustration of the multi-step stopping mechanism in DeconX. The illustration is created with Biorender.com. (b) Residual norm across iterations for scenarios with 0, 1, or 2 missing cell types. (c) Stopping accuracy as a function of signature similarity threshold ($t_{1}$) and marker gene uniqueness threshold ($t_{2}$). (d) Ablation analysis comparing stopping accuracy when using signature similarity ($t_{1}$) alone, marker gene uniqueness ($t_{2}$) alone, or both criteria combined ($t_{1}$ & $t_{2}$).

DeconX employs a multi-step stopping mechanism to determine when to terminate the iterative discovery process (Fig. 4a). After the first iteration, the algorithm evaluates whether the residual norm shows significant reduction compared to the initial state. If no significant reduction is observed, the algorithm concludes that no missing cell types are present and terminates immediately. If significant reduction is detected after the first iteration, DeconX proceeds to iteratively identify additional missing cell types, evaluating two quality criteria for each newly predicted signature: (1) the maximum similarity between the predicted signature and all previously discovered missing cell type signatures must be below threshold $t_{1}$, and (2) the uniqueness of marker genes, defined as one minus the overlap ratio between the marker genes of the newly predicted signature and those of previously discovered missing cell types, must exceed threshold $t_{2}$. The algorithm stops if either criterion is violated, indicating that the predicted signature likely represents noise or redundant signal rather than a distinct missing cell type.

We validated these stopping criteria on simulated data through systematic evaluation across 13 simulation scenarios, including 1 case with 0 missing cell types and 4 cases each for 1, 2, and 3 missing cell types, respectively. Tracking residual norms confirmed that scenarios with missing cell types showed rapid decline after the first iteration, while scenarios without missing cell types showed minimal reduction, validating the first-round check (Fig. 4b). We then optimized the quality thresholds by varying $t_{1}$ from 0.5 to 0.7 and $t_{2}$ from 20% to 60%, achieving optimal stopping accuracy of 0.69 (Fig. 4c). Ablation analysis demonstrated that combining both quality criteria ($t_{1}$ and $t_{2}$) achieved substantially higher stopping accuracy compared to using either criterion alone, confirming their complementary roles in termination decisions (Fig. 4d).

## 4 Discussion

DeconX addresses a critical gap in current deconvolution approaches caused by incomplete single-cell references, where certain cell types present in bulk samples are absent from the reference atlas. Through systematic evaluation on simulated and real bulk RNA-seq data, we demonstrated that DeconX uniquely offers the capability to identify the proportion, signatures, and number of reference-missing cell types from bulk transcriptomics datasets. Several future directions could extend DeconX’s capabilities. First, integrating spatial transcriptomics data could enable spatially-aware deconvolution and discovery. Second, the iterative discovery mechanism could be further enhanced to improve detection of missing cell types that share high transcriptional similarity with existing reference cell types, which currently remain challenging to identify. Finally, incorporating large language model agents could automate the discovery workflow, from adaptive parameter selection to biological interpretation of discovered cell types, making the framework more accessible to researchers without extensive computational expertise.

## Supplementary Material

btag412_Supplementary_Data

## Data Availability

This study did not generate new primary sequencing data. The simulated pseudo-bulk analyses used publicly available COVID-19 PBMC single-cell RNA-seq data, for which the processed data are available from ArrayExpress under accession E-MTAB-10026 and the h5ad data object is available through the COVID-19 Cell Atlas (https://covid19cellatlas.org). The HGSOC analyses used publicly available paired bulk RNA-seq, scRNA-seq and snRNA-seq data. Processed gene count tables for this dataset are available in GEO under accession GSE217517, and the corresponding raw FASTQ files are available through dbGaP under accession phs002262.v2.p1. Any pseudo-bulk datasets analysed in this study were computationally generated from these public datasets as described in the Methods.

## Author contribtions

Yimin Fan (Conceptualization, Funding acquisition, Methodology, Software, Formal analysis, Investigation, Project administration, Visualization, Writing—original draft, Writing—review & editing,), Yixuan Liu (Methodology, Formal analysis, Investigation, Visualization, Writing—original draft), Yunhua Zhong (Methodology, Formal analysis, Investigation, Visualization), Yue Wang (Methodology, Investigation, Data curation, Validation), Kin Hei Lee (Investigation, Methodology, Data curation, Validation), Yixuan Wang (Investigation, Writing—review & editing), Xinyuan Liu (Investigation, Writing—review & editing), Jiayi Li (Investigation, Writing—review & editing), Xuesong Wang (Investigation, Writing—review & editing), Ziqian Lin (Investigation, Validation, Writing—review & editing), Lei Li (Conceptualization, Supervision, Project administration, Resources, Funding acquisition), and Yu Li (Conceptualization, Supervision, Project administration, Resources, Funding acquisition)
