# Supplementary material for: Discovering reference-missing cell types in bulk transcriptomics
Source: Bioinformatics. 2026 Aug 21;42(Suppl 2):btag412. doi: 10.1093/bioinformatics/btag412 (PMC13501327; doi:10.1093/bioinformatics/btag412)
Supplement: Supplementary file 1 [file btag412_supplementary_data.pdf]

Journal Title Here, YEAR, , 1–14
DOI added during production
Published: Date added during production
Paper

Supplementary Materials for Discovering Reference-Missing Cell Types in Bulk Transcriptomics

Yimin Fan,[1,*,†] Yixuan Liu,[1,*] Yunhua Zhong,[1,*] Yue Wang,[1] Kin Hei Lee,[1] Yixuan Wang,[1] Xinyuan Liu,[1] Jiayi Li,[1] Xuesong Wang,[1] Ziqian Lin,[1] Lei Li[2,†] and Yu Li[1,†]

[1]Department of Computer Science and Engineering, The Chinese University of Hong Kong, HKSAR, China
[2]Canchen Technology, Shenzhen, China
[*]These authors contributed equally to this work.[†]Corresponding author. fanyimin@link.cuhk.edu.hk, levi.li@novaeve.ai, liyu@cse.cuhk.edu.hk

Abstract

These are the supplementary notes, figures and tables.

1. Supplementary Notes

1.1. Runtime and GPU memory benchmark

We recorded the running time and resource consumption of DeconX in end-to-end runs on both simulated and real bulk datasets. The experimental configuration is summarized in Supplementary Table 1, and the detailed wall-clock runtime and peak GPU memory usage are shown in Supplementary Figure 9.

On simulated PBMC bulk data, the wall-clock runtime increased approximately linearly with the number of bulk samples, denoted by n. The mean runtime across three trials was 23.5 min at $n = 100$, 36.8 min at $n = 1000$, 66.6 min at $n = 3000$, and 96.9 min at $n = 5000$. Individual trials for each n are shown as dots in Supplementary Figure 9A. These results indicate that DeconX remains computationally efficient for large-scale bulk deconvolution.

To benchmark runtime on a multi-missing-cell-type setting, we constructed an HGSOC-based simulation in which four cell types—plasma cells, adipocytes, macrophages, and mesothelial cells—were held out from the reference. On this benchmark with 24 bulk samples, DeconX completed the full pipeline in 25.2 min, with the cumulative breakdown across rounds shown in Supplementary Figure 9B. This runtime indicates that DeconX remains efficient on real bulk datasets.

On simulated PBMC bulk data, peak GPU memory increased slowly with the number of bulk samples n, from 261 MB at $n = 100$ to 574 MB at $n = 5000$. The peak memory usage for each tested sample size is shown in Supplementary Figure 9C. Even at the largest tested sample size, DeconX used less than 600 MB of GPU memory, suggesting that the method has a modest resource requirement and can be readily used in standard GPU environments.

1.2. Overview of simulation scenarios

All simulation experiments were based on the COVID-19 PBMC single-cell atlas (Stephenson et al., 2021). Pseudo-bulk mixtures were generated using a Dirichlet-based single-cell sampling procedure, similar to the simulation strategy used in TAPE (Chen et al., 2022). For each pseudo-bulk sample, cell-type proportions were sampled from a Dirichlet distribution with cell-type-specific concentration parameters. The concentration parameters of the known reference cell types were fixed at $\alpha = 2.0$. For the candidate missing cell type, the concentration parameter was varied over $\alpha_{\text{miss}} \in \{0.5, 1.0, 2.0\}$ to generate different missing-cell-type abundance levels.

Given the sampled cell-type proportions, 3,000 single cells were sampled with replacement from the corresponding annotated cell-type subsets, with the number of cells assigned to each cell type determined by the sampled proportions. The expression profiles of the sampled cells were then summed to obtain one pseudo-bulk expression profile.

Across all simulations, the known cell types provided to DeconX were fixed as B_cell, CD4, CD8, and CD14. To evaluate the ability of DeconX to capture signals from cell types absent from the observed reference, we considered the following candidate missing cell types: RBC, DCs, Lymph_prolif, CD16, Plasmablast, Platelets, pDC, Mono_prolif, MAIT, gdT, HSC, Treg, NK_16hi, and NK_56hi. In each missing-cell-type scenario, the selected candidate cell types were excluded from the reference provided to DeconX while still being included in the pseudo-bulk generation process.

1.3. Rationale and validation of residual-based inference

DeconX uses residual expression after reference-based deconvolution to identify signals that are not explained by the observed reference cell types. Here, we clarify the mathematical rationale for this interpretation and provide empirical support using both real HGSOC bulk data and simulated PBMC pseudo-bulk data.

Let $b_j \in \mathbb{R}^G$ denote the expression profile of bulk sample j, and let $S \in \mathbb{R}^{K \times G}$ denote the reference signature matrix containing K observed cell types, where $s_i \in \mathbb{R}^G$ is the signature vector of reference cell type i. If all cell types in the bulk

Received: Date Month Year. **Revised received:** Date Month Year. **Accepted:** Date Month Year
© The Author(s) YEAR. Copyright and licence statements to be updated by the publisher during production.

sample are represented in the reference, the bulk profile can be approximated as

$$b_j = \sum_{i=1}^{K} p_{ij} s_i + \epsilon_j,$$

where p_{ij} is the proportion of reference cell type i, and ϵ_j denotes technical noise.

When M cell types are missing from the reference, the true generative model can be written as

$$b_j = \sum_{i=1}^{K} p_{ij} s_i + \sum_{m=1}^{M} q_{mj} u_m + \epsilon_j,$$

where $u_m \in \mathbb{R}^G$ and q_{mj} denote the expression signature and proportion of missing cell type m, respectively. Standard deconvolution estimates $\hat{p}_{ij}$ using only the observed reference cell types, yielding the residual

$$\mathrm{res}_j = b_j - \sum_{i=1}^{K} \hat{p}_{ij} s_i.$$

Thus, the residual represents the component of the bulk signal not explained by the observed reference. When the missing cell types have transcriptional signals that are not fully captured by the observed reference cell types, the residual can retain missing-cell-type-specific components, together with technical noise and model-mismatch effects. This interpretation does not assume that the residual is identical to the complete missing-cell-type expression profile; rather, it motivates using residuals as an information source for discovering missing cell-type signals.

We empirically evaluated this interpretation in DeconX Round 0 using both real HGSOC bulk data and simulated PBMC pseudo-bulk data. To distinguish the signal directly present in the residuals from the signature reconstructed by DeconX, we analyzed two residual-derived representations:

- **Raw residual**: the unexplained expression component after fitting bulk samples using only the observed reference cell types.
- **DeconX-inferred signature**: the expression signature reconstructed by DeconX from the residual signals to represent the missing cell type.

For both representations, we performed cosine-similarity ranking against candidate cell-type signatures. We also performed a marker-level recovery analysis to test whether known markers of the held-out cell type were enriched in the residual, using markers of a present reference cell type as a specificity control.

In the real HGSOC bulk data with 24 patient samples, adipocytes and plasma cells were held out from the reference, and Plasma cells were evaluated as the target missing cell type. The raw residual ranked Plasma cells first among 13 candidate signatures, with cosine similarity 0.418, followed by the next-ranked cell type at 0.392. The DeconX-inferred signature also ranked Plasma cells first, with cosine similarity 0.421, while the next-ranked cell type decreased to 0.357. These results indicate that the held-out Plasma cell signal was present in the residual and became more separated from other candidate cell types after DeconX reconstruction.

In the simulated PBMC pseudo-bulk data with known ground truth, Plasmablast was held out from the reference. The raw residual ranked Plasmablast first among 16 candidate signatures, with cosine similarity 0.865, followed by the next-ranked cell type at 0.347. The DeconX-inferred signature also ranked Plasmablast first, with cosine similarity 0.865, followed by the next-ranked cell type at 0.346. These results show that missing-cell-type signal was strongly retained in the residuals in a controlled setting with known ground truth.

Using the same simulated Plasmablast holdout setting, we further evaluated whether the residual was enriched for known markers of the missing cell type. Plasmablast markers were selected from PanglaoDB "Plasma cells" (Franzén et al., 2019), including *IGHG1*, *IGHG2*, *IGKC*, *IGLC3*, *IGHM*, *MZB1*, and *CD38*. These markers had substantially higher mean expression in the residual than in the reference baseline, with residual 12.52 versus baseline 0.36, corresponding to a 35-fold enrichment.

As a specificity control, we examined CD4 T-cell markers selected from PanglaoDB "T cells" (Franzén et al., 2019) and Sallusto et al. (Sallusto et al., 1999), including *IL7R*, *CCR7*, *CD4*, *TCF7*, *LEF1*, *TNFRSF4*, and *SELL*. These markers correspond to a cell type already present in the reference and were not spuriously elevated in the residual. Their mean expression was lower in the residual than in the reference baseline, with residual 1.23 versus baseline 2.68.

Together, the cosine-similarity and marker-level analyses support the interpretation that residuals can retain structured, cell-type-specific signals from held-out cell types rather than representing only arbitrary unexplained variation.

1.4. Benchmark against residual NMF for missing-cell-type signature recovery

We further benchmarked DeconX against a residual NMF baseline to assess whether missing-cell-type signals could be recovered from deconvolution residuals by matrix factorization alone. The comparison was performed on both simulated PBMC pseudo-bulk data and the HGSOC real-bulk dataset. For each dataset, the same bulk expression matrix and reference single-cell expression data were used for DeconX and the residual NMF baseline.

For the residual NMF baseline, we first performed reference-based deconvolution using the incomplete reference and then applied non-negative matrix factorization to the residual expression matrix. Following the residual factorization strategy described by Ivich et al. (Ivich et al., 2025), the residual matrix was factorized into three components. Because NMF components have arbitrary ordering and do not directly correspond to predefined cell types, we used a post-hoc matching strategy for evaluation: for each held-out or unobserved cell type, we selected the NMF component with the highest enrichment of its canonical marker genes. This selection was used only for evaluation and therefore represents a favorable setting for the residual NMF baseline.

To quantify missing-cell-type signature recovery, we computed a marker-vs-non-marker enrichment fold for each method. Let s denote the method-specific per-gene score, defined as the selected NMF component profile for the residual

NMF baseline and as the DeconX-inferred missing-cell-type signature for DeconX. All scores were restricted to the shared gene set G between the bulk and reference data. For a canonical marker set $M \subset G$, with background genes $\bar{G} = G \setminus M$, the enrichment fold was defined as

$$\text{fold} = \frac{(1/|M|)\sum_{g\in M} s_g}{(1/|\bar{G}|)\sum_{g\in \bar{G}} s_g}.$$

A fold of 1 indicates no enrichment of canonical markers over background genes, whereas larger values indicate stronger recovery of the missing-cell-type signature. Marker genes were not provided to either method and were used only for post-hoc validation.

In simulated PBMC pseudo-bulk data, plasmablasts and dendritic cells (DCs) were held out from the reference. Residual NMF achieved marker-vs-non-marker folds of 2.29 for plasmablasts and 1.02 for DCs, whereas DeconX achieved folds of 71.3 and 9.8, respectively. In the HGSOC real-bulk dataset, plasma cells and adipocytes were held out from the reference. Residual NMF achieved folds of 1.09 for plasma cells and 1.98 for adipocytes, whereas DeconX achieved folds of 7.8 and 65.5, respectively. These results show that DeconX more effectively recovers biologically interpretable missing-cell-type signatures than residual NMF across both simulated and real-bulk benchmarks (Supplementary Figure 6).

1.5. Sensitivity analysis of the PCA top-% threshold

To assess the robustness of DeconX to the sample-selection threshold used in the residual PCA space, we varied the PCA top-% threshold from 5% to 20% in both real and simulated datasets. For each threshold, samples with the highest PC1 scores were selected as residual-enriched samples and used for pseudo-cell profile estimation.

We evaluated the recovered missing-cell-type signal using two complementary metrics. First, marker gene enrichment was calculated as the fold increase in the average expression of known target cell-type markers in PCA-selected samples relative to non-selected samples. Second, cosine similarity was calculated between the recovered pseudo-cell profile and the corresponding target cell-type profile.

Across the tested thresholds, DeconX maintained clear marker gene enrichment and stable cosine similarity in both real and simulated datasets (Supplementary Figure 7). These results indicate that the recovered missing-cell-type signal is robust to reasonable choices of the PCA top-% threshold. Smaller thresholds select the most extreme samples in the residual PCA space and may therefore favor stronger and more specific missing-cell-type signals, whereas larger thresholds include more samples but may also introduce weaker enrichment or additional residual heterogeneity.

1.6. Functional enrichment analysis of the DeconX-recovered adipocyte signature

To ensure a systematic and transparent interpretation of the functional enrichment results, we performed pre-ranked gene set enrichment analysis (GSEA) using `gseapy.prerank`. Genes were ranked by the residual-derived adipocyte signature score, defined as

$$\log_2(\text{DeconX-recovered adipocyte signature}+1) - \log_2(\text{real bulk mean}+1),$$

where larger values indicate stronger enrichment in the DeconX-recovered adipocyte signature relative to the bulk background.

We tested 1,883 Gene Ontology Biological Process (GO-BP) gene sets using `min_size=15`, `max_size=500`, and `permutation_num=1000`. For each gene set, we reported the enrichment score (ES), normalized enrichment score (NES), nominal p-value, and false discovery rate (FDR). Gene sets with FDR < 0.25 were considered significant according to the conventional GSEA threshold.

In total, seven GO-BP terms passed the FDR < 0.25 threshold, including six positively enriched metabolic processes and one negatively enriched immune-related process (Supplementary Figure 5). Positive enrichment indicates that the gene set is enriched at the high-ranking end of the DeconX-recovered adipocyte signature, whereas negative enrichment indicates enrichment at the low-ranking end. Because our goal was to evaluate whether the recovered signature captured adipocyte-associated biology, we focused the interpretation on the positively enriched terms.

1.7. Robustness to epithelial subtype heterogeneity in HGSOC

HGSOC tumors can exhibit substantial epithelial-cell heterogeneity, which may reflect tumor-associated molecular states not captured by broad cell-type annotations. To evaluate whether such heterogeneity affects DeconX, we further stratified the epithelial compartment in the HGSOC single-cell reference into molecular subtypes and repeated the missing-cell-type recovery analysis under different reference configurations.

We classified epithelial cells into HGSOC molecular subtypes using established subtype marker genes. Specifically, subtype scores were computed for each epithelial cell using marker-gene sets for the differentiated (DIF) and mesenchymal (MES) subtypes. Cells with a dominant subtype score were assigned to the corresponding subtype, whereas the remaining epithelial cells were retained as unspecified epithelial cells. This resulted in three epithelial labels: Epithelial_DIF, Epithelial_MES, and unspecified Epithelial.

We then constructed four reference configurations that differed only in the inclusion of the epithelial subtypes: unspecified epithelial cells only, unspecified epithelial cells with DIF epithelial cells, unspecified epithelial cells with MES epithelial cells, and unspecified epithelial cells with both DIF and MES epithelial cells. DeconX was applied to each configuration using the same bulk RNA-seq data, and the recovered missing signature was compared with the adipocyte profile derived from the matched snRNA-seq reference.

Across all four configurations, DeconX consistently recovered adipocytes as the missing cell type. The cosine similarity between the recovered adipocyte pseudo-signature and the reference adipocyte profile remained stable across configurations, with values of 0.444, 0.437, 0.438, and 0.441, respectively. Moreover, the DIF and MES epithelial

subtypes were not falsely identified as missing cell types. These results indicate that DeconX is robust to epithelial subtype heterogeneity in this HGSOC dataset.

2. Supplementary Tables

Supplementary Table 1. DeconX experimental configuration.

Parameter	Value
GPU	NVIDIA A100-SXM4-40GB
CPU cores	4
RAM	150 GB
Epochs	200
Batch size	256
Learning rate	10^{-3}

3. Supplementary Figures

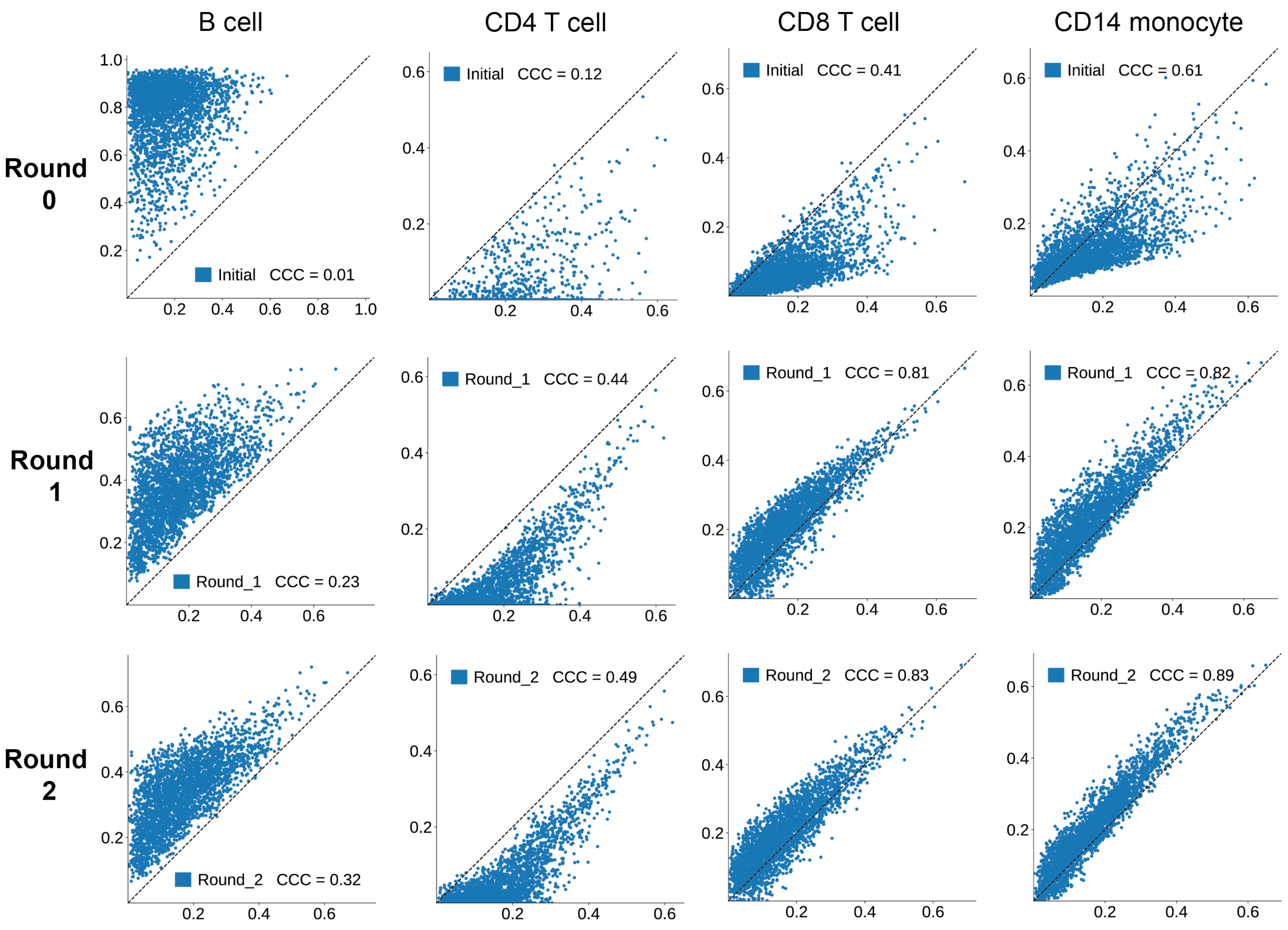

Supplementary Figure 1 Iterative improvement of proportion predictions across DeconX rounds with two missing cell types (Plasmablasts, DC cells). Scatter plots show predicted versus ground-truth proportions for four reference cell types (B cells, CD4 T cells, CD8 T cells, and CD14 monocytes) at different stages: Round 0 (initial deconvolution without accounting for missing cell types), Round 1 (after identifying the first missing cell type), and Round 2 (after identifying the second missing cell type). CCC values improved progressively across rounds for all reference cell types, demonstrating that iteratively incorporating discovered missing cell types enhances deconvolution accuracy. This multi-round process enables DeconX to handle scenarios with multiple missing cell types by sequentially detecting and inferring their proportions and expression signatures.

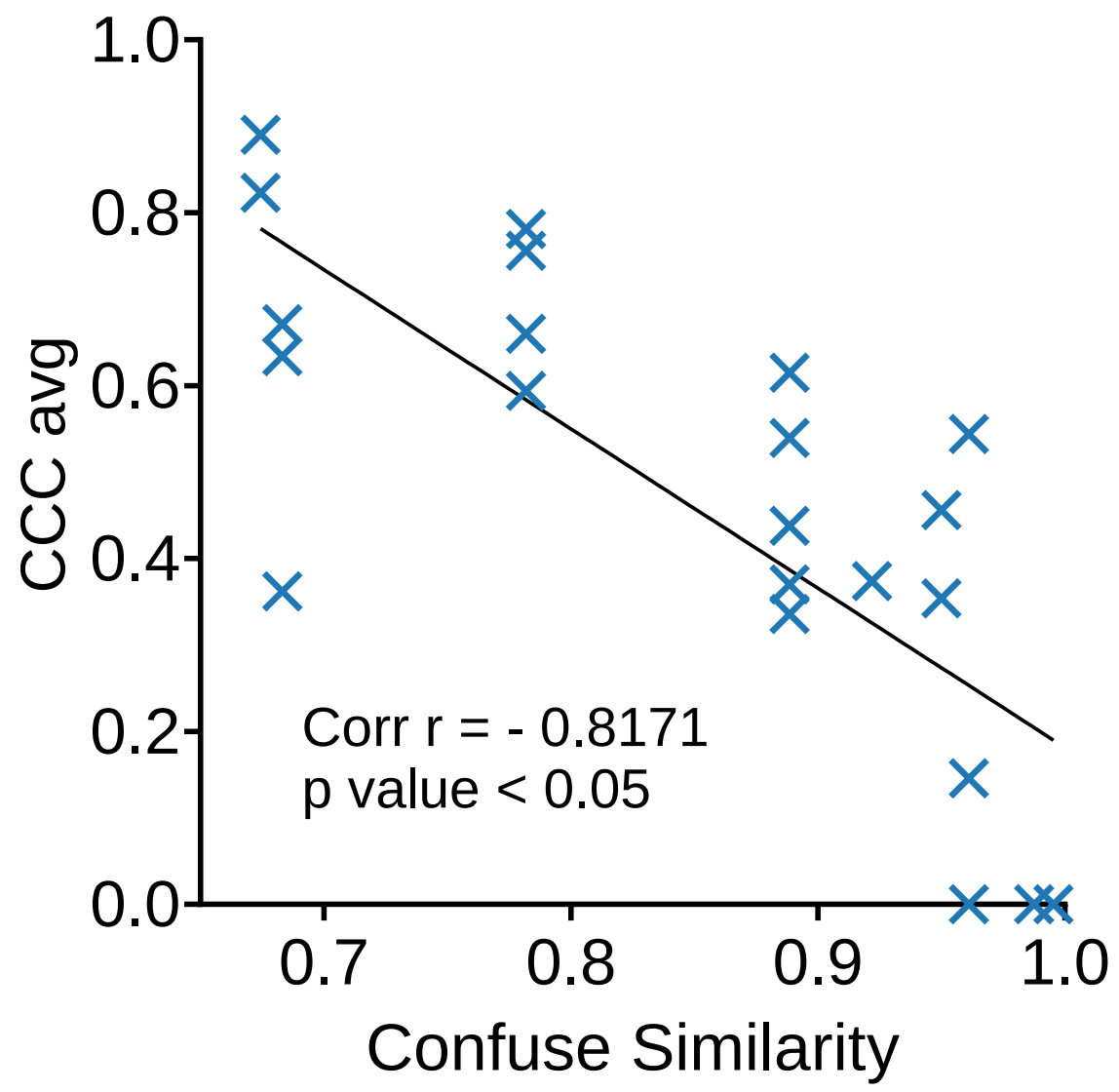

Supplementary Figure 2 Relationship between the confuse similarity (maximum missing cell type signature similarity with the reference cell types) and the average CCC scores. Higher similarity leads to lower performance in predicting the cell type proportion. Corr indicates spearman correlation coefficient.

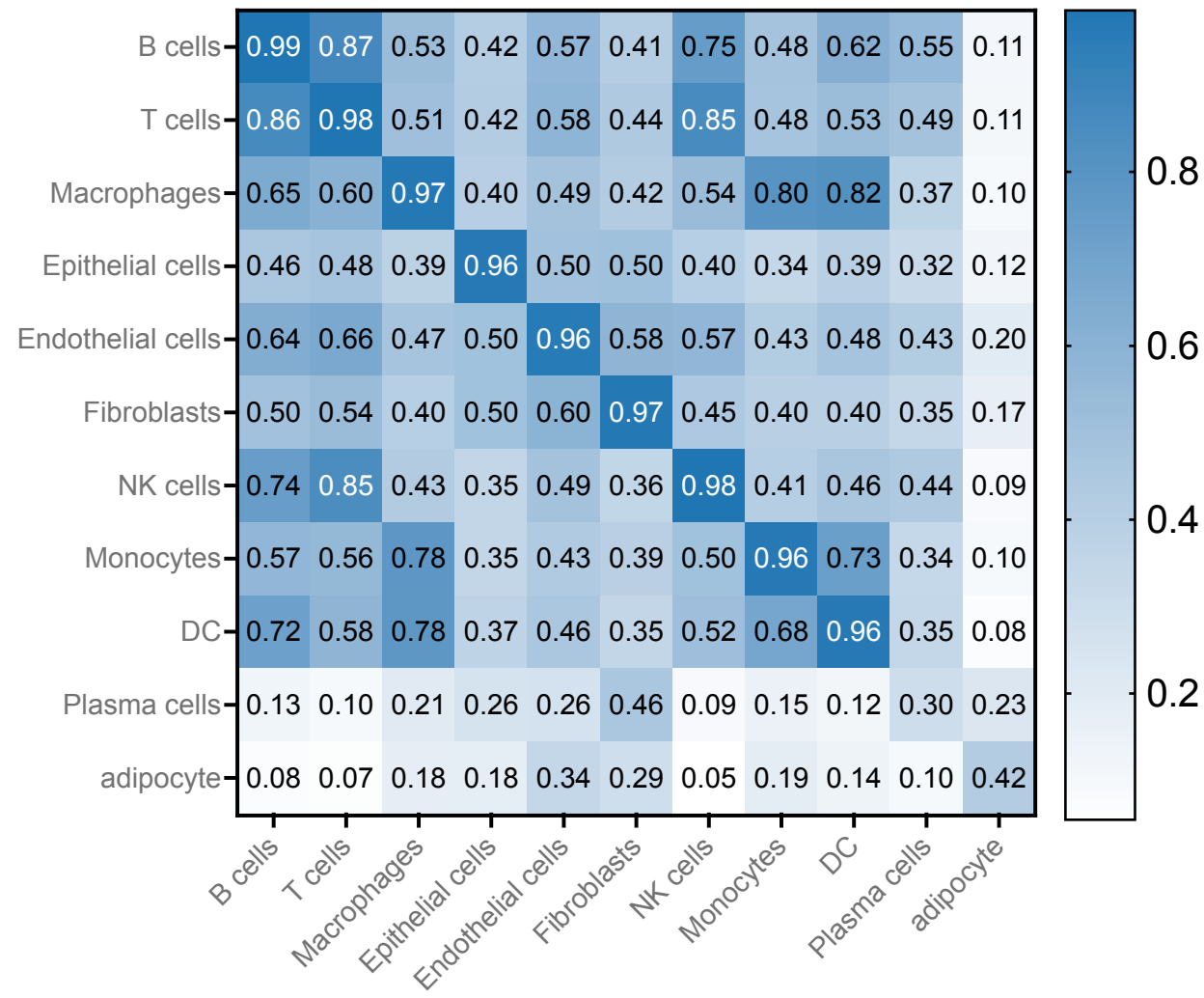

Supplementary Figure 4 Cosine similarity between DeconX-reconstructed signatures and snRNA-seq-derived cell-type profiles in the HGSOC setting. Rows denote DeconX output signatures after model updating, and columns denote reference cell-type profiles from the matched snRNA-seq data. The recovered missing signatures showed highest similarity to their corresponding snRNA-seq-derived cell-type profiles.

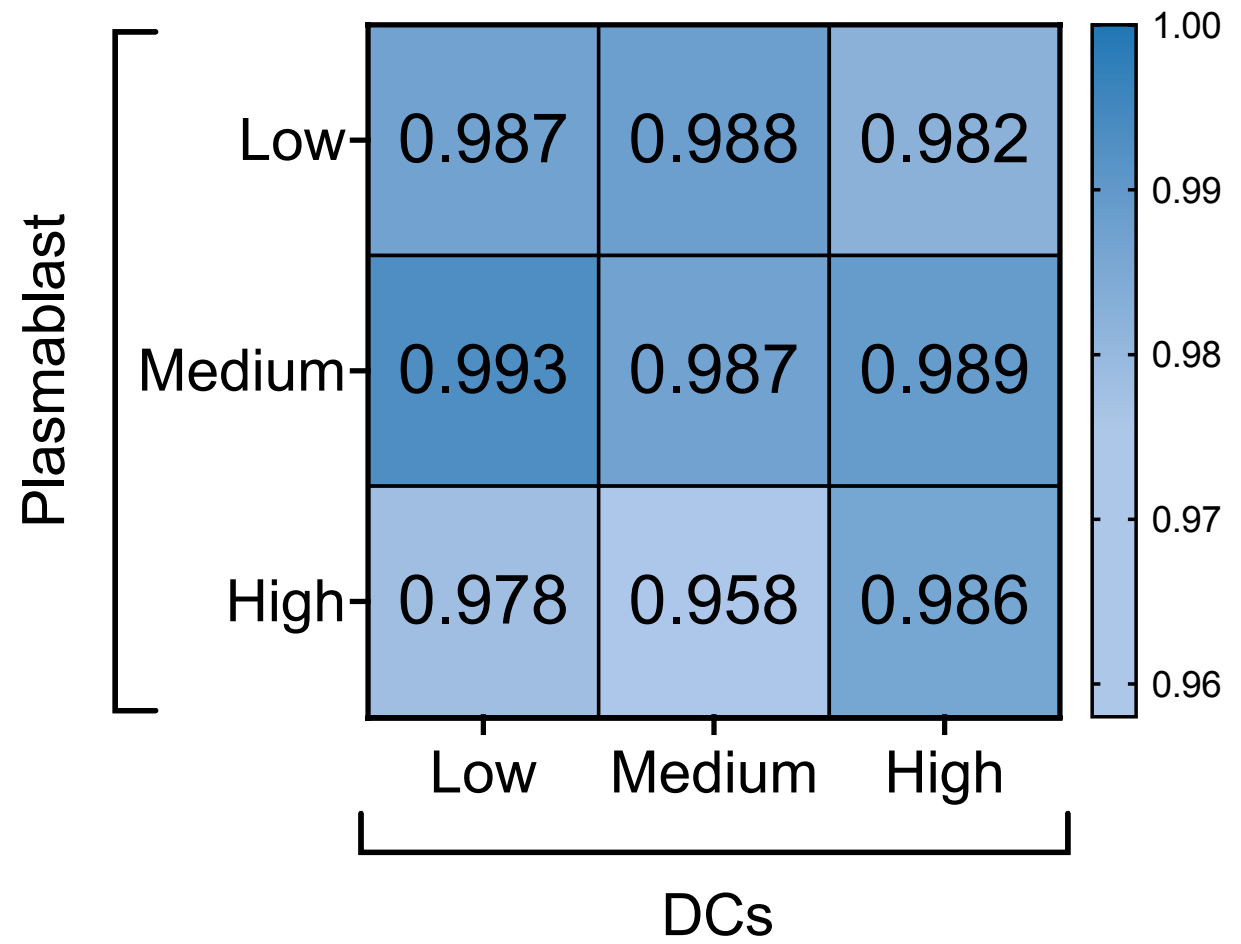

Supplementary Figure 3 Proportion CCC performance for Plasmablast cell with two missing cell types at different proportion levels.

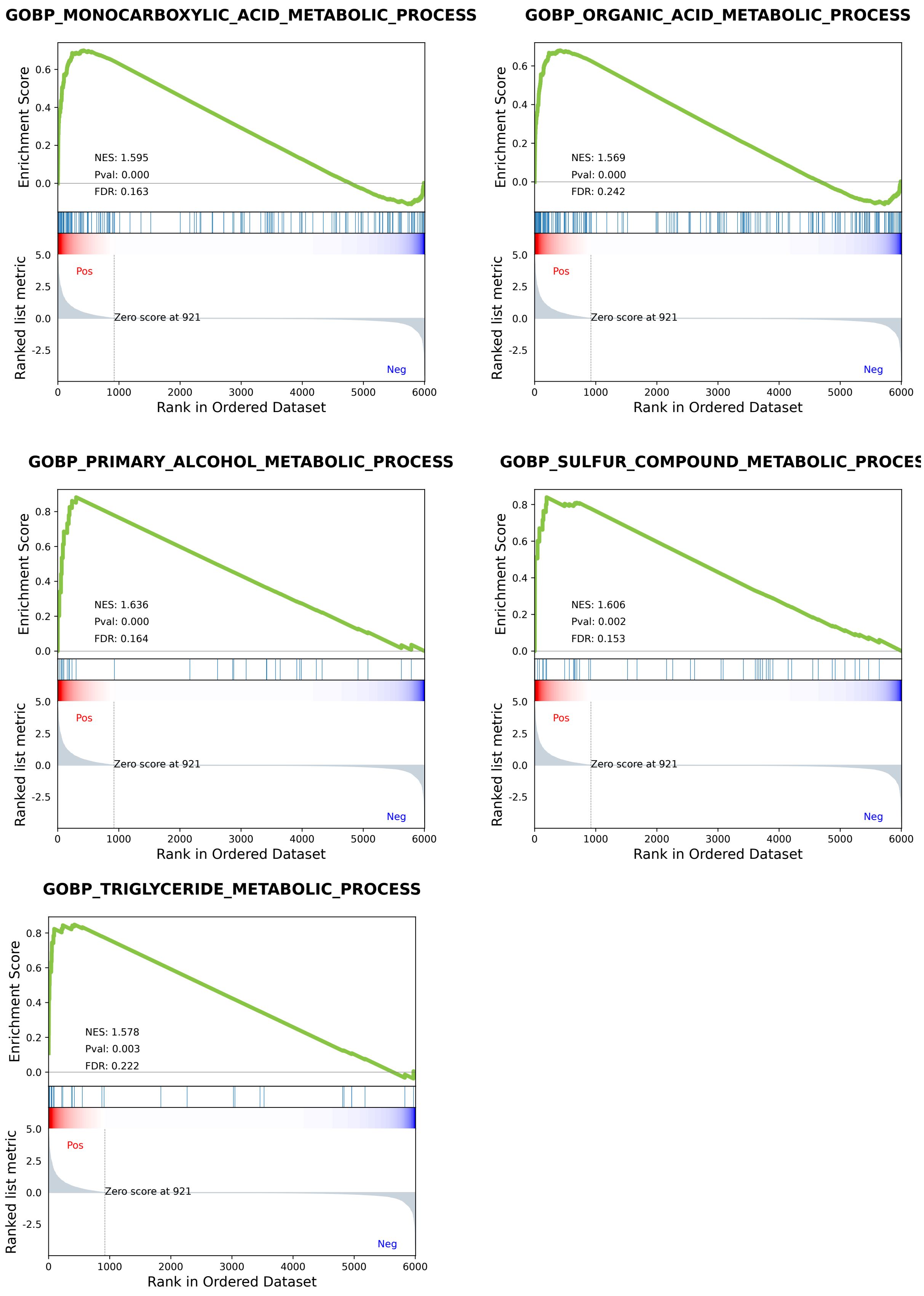

Supplementary Figure 5 The pathway enrichment analysis of the residual on real datasets.

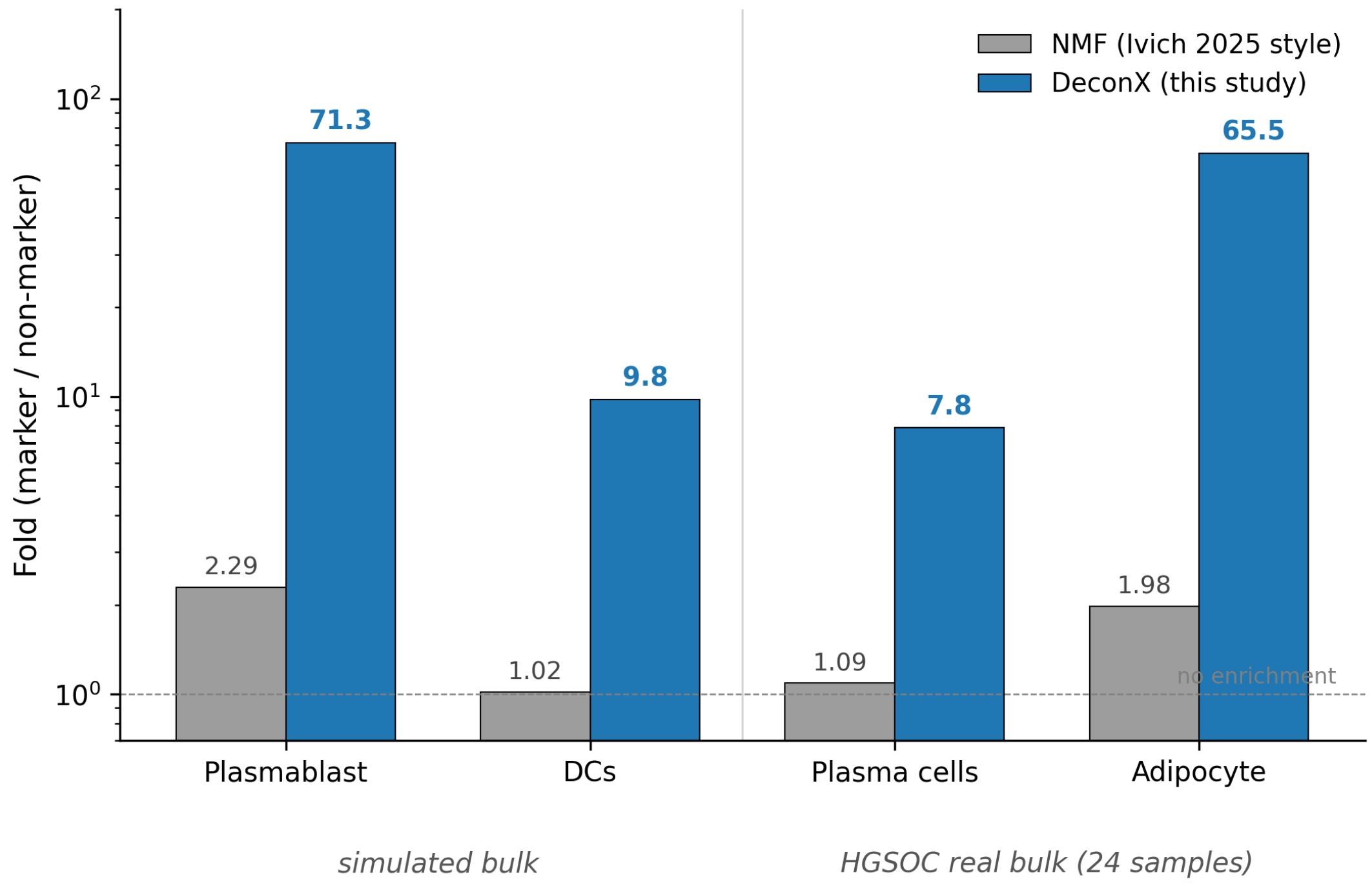

Supplementary Figure 6 Comparison between DeconX and residual NMF for recovering missing cell-type signatures. Marker enrichment was measured as the average marker-gene score relative to the average non-marker-gene score; higher values indicate stronger recovery of the missing cell-type signal. Results are shown for simulated PBMC pseudo-bulk data with plasmablasts and DCs held out, and for HGSOC real-bulk data with plasma cells and adipocytes missing from the reference.

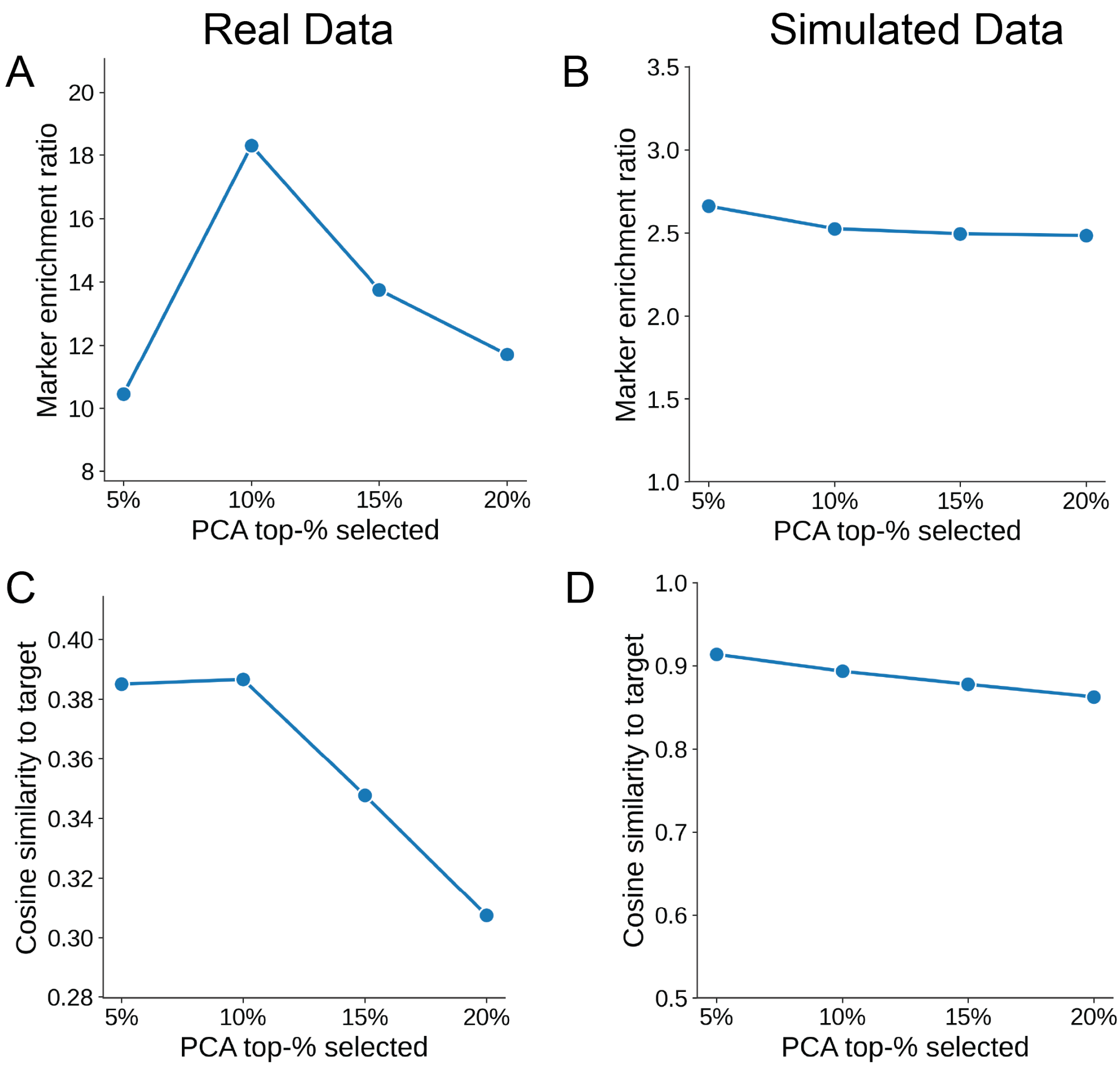

Supplementary Figure 7 Sensitivity analysis of the PCA top-% sample-selection threshold. The PCA top-% threshold was varied from 5% to 20% to evaluate its effect on missing-cell-type signal recovery. **A–B.** Marker-enrichment ratios in the real (**A**) and simulated (**B**) datasets. **C–D.** Cosine similarity between the recovered pseudo-cell and target cell-type profiles in the real (**C**) and simulated (**D**) datasets. Marker enrichment was defined as the fold increase in average target-marker expression in PCA-selected samples relative to non-selected samples.

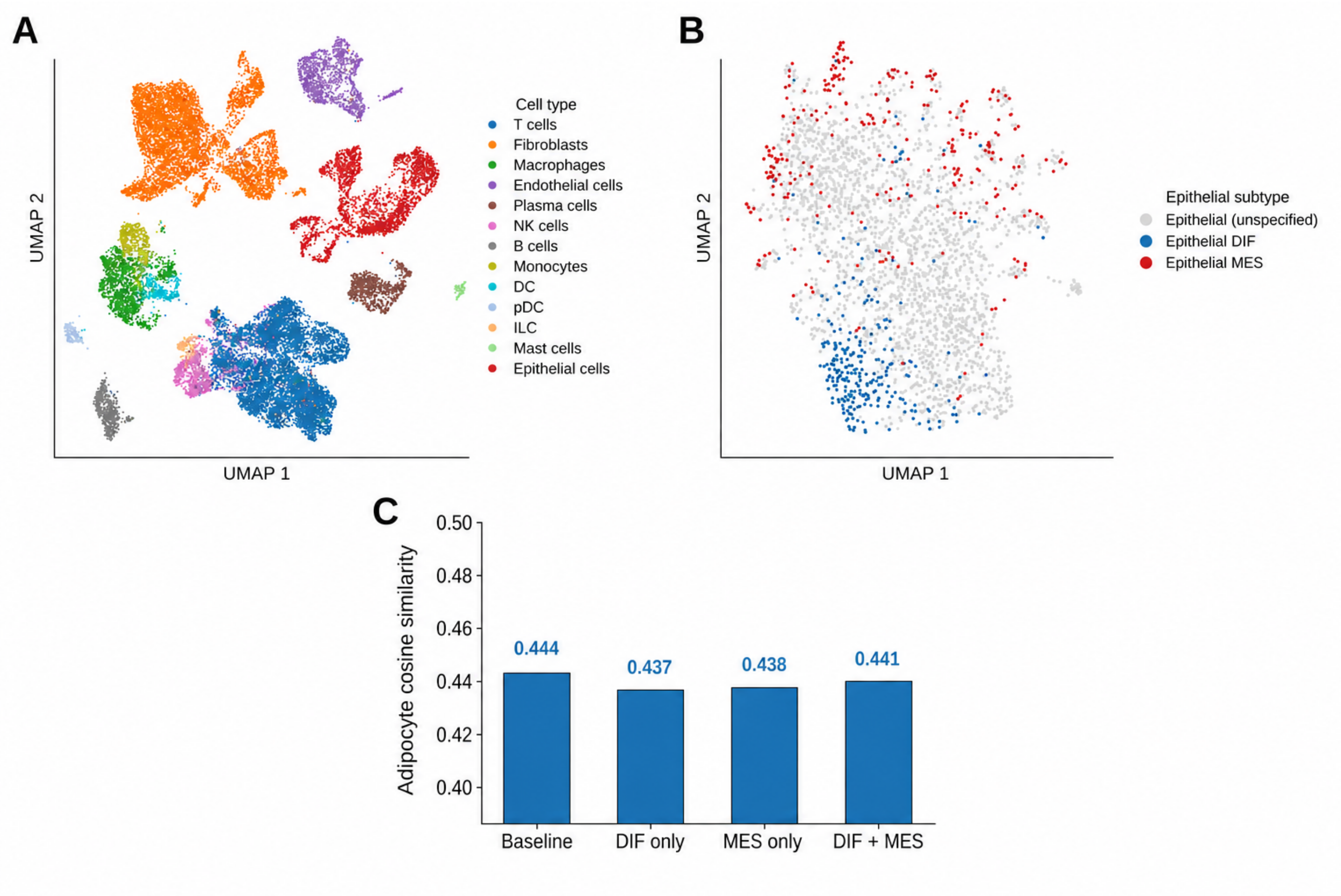

Supplementary Figure 8 DeconX applied to the HGSOC tumor dataset. **A.** UMAP of all HGSOC scRNA-seq cells, coloured by annotated cell type. **B.** UMAP of the epithelial (tumor) compartment alone, coloured by the inferred molecular subtype (Differentiated, Mesenchymal, or unspecified epithelial). **C.** Cosine similarity between the DeconX-recovered adipocyte pseudo signature and the reference adipocyte profile, under four reference configurations that differ only in the representation of the epithelial compartment: epithelial cells only (Baseline), epithelial cells plus the DIF subtype, plus the MES subtype, and plus both subtypes. The recovered adipocyte signature is essentially unchanged across all four settings.

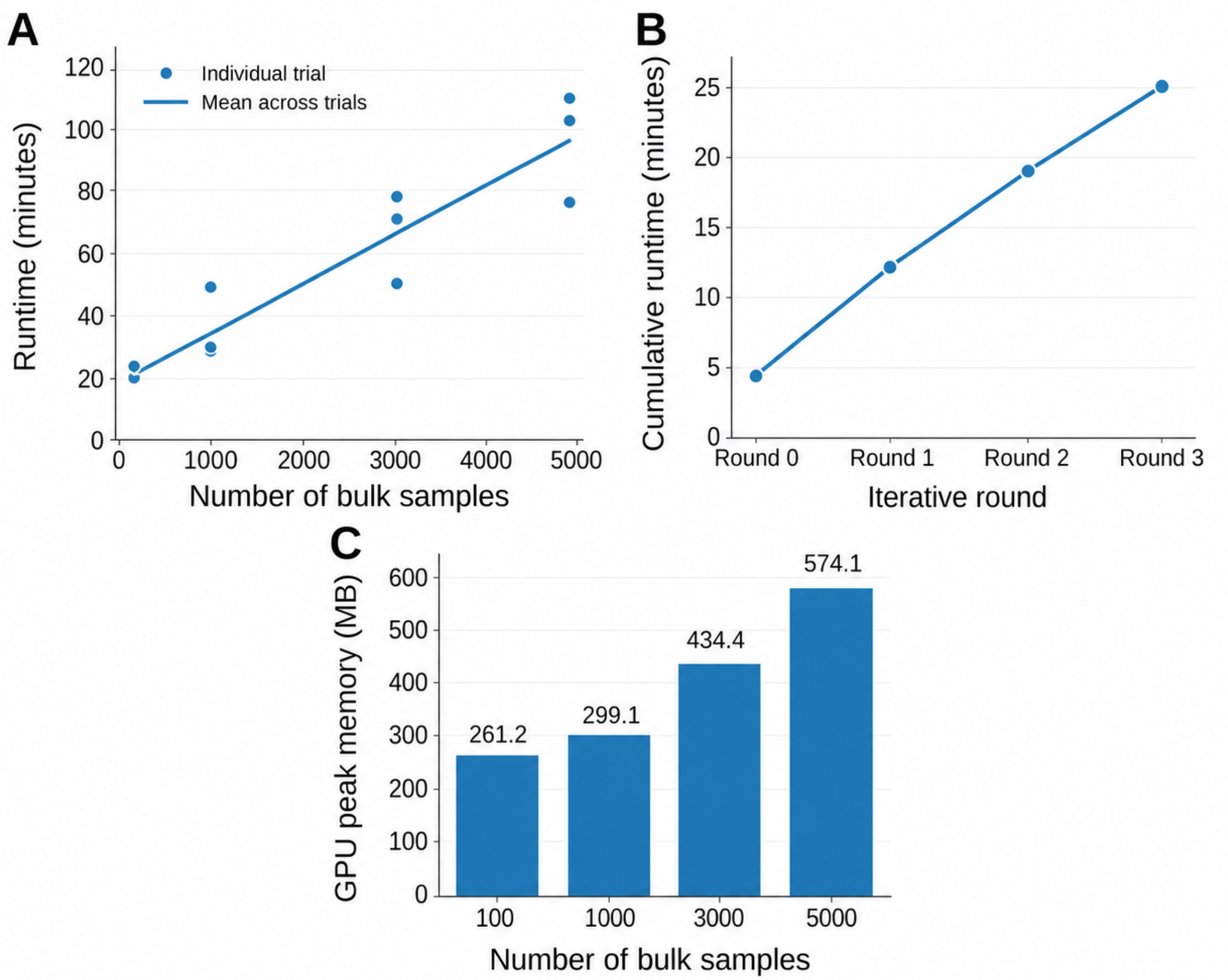

Supplementary Figure 9 DeconX runtime and GPU memory benchmark. **A.** Wall-clock runtime versus the number of bulk samples n on simulated PBMC bulk data, using a reference in which plasmablasts and dendritic cells were held out. Dots indicate individual trials for each n, and the line connects the mean runtime across three trials at each tested n. **B.** Cumulative wall-clock runtime across discovery rounds on real HGSOC bulk data from 24 patient samples, where DeconX inferred four cell types missing from the reference: plasma cells, adipocytes, macrophages, and mesothelial cells. **C.** Peak GPU memory versus the number of bulk samples n on the same simulated PBMC benchmark; numerical values are annotated above each bar. Hardware and pipeline configurations are listed in Supplementary Table 1.

References

Y. Chen, Y. Wang, Y. Chen, Y. Cheng, Y. Wei, Y. Li, J. Wang, Y. Wei, T.-F. Chan, and Y. Li. Deep autoencoder for interpretable tissue-adaptive deconvolution and cell-type-specific gene analysis. *Nature Communications*, 13(1):6735, 2022.

O. Franzén, L.-M. Gan, and J. L. Björkegren. Panglaodb: a web server for exploration of mouse and human single-cell rna sequencing data. *Database*, 2019:baz046, 2019.

A. Ivich, N. R. Davidson, L. Grieshober, W. Li, S. C. Hicks, J. A. Doherty, and C. S. Greene. Missing cell types in single-cell references impact deconvolution of bulk data but are detectable. *Genome biology*, 26(1):86, 2025.

F. Sallusto, D. Lenig, R. Förster, M. Lipp, and A. Lanzavecchia. Two subsets of memory t lymphocytes with distinct homing potentials and effector functions. *Nature*, 401(6754):708–712, 1999.

E. Stephenson, G. Reynolds, R. A. Botting, F. J. Calero-Nieto, M. D. Morgan, Z. K. Tuong, K. Bach, W. Sungnak, K. B. Worlock, M. Yoshida, et al. Single-cell multi-omics analysis of the immune response in covid-19. *Nature medicine*, 27(5): 904–916, 2021.
